# Supplementary material for: Estimates of pandemic excess mortality in India based on civil registration data
Source: PLOS Glob Public Health. 2022 Dec 9;2(12):e0000803. doi: 10.1371/journal.pgph.0000803 (PMC10021303; doi:10.1371/journal.pgph.0000803)
Supplement: S1 Text — (DOCX) [file pgph.0000803.s001.docx]

**S1Text:** Supporting Information on death registration completion and additional methodological notes

**Estimates of crude death rate and registration completion in 2019**

The 2019 report on Vital Statistics of India based on the Civil Registration System [1], provides both national and sub-national data on registered deaths, along with estimates of completion. The latter draw on estimates of the crude death rate (CDR) from the 2018 Sample Registration System annual statistical report [2]. Recently, the 2019 Sample Registration System annual statistical report [3] has also been made available. Completion nationally or sub-nationally can be estimated by comparing total registered deaths to total expected deaths based on CDR and population estimates.

The [2018 SRS report](https://censusindia.gov.in/Vital_Statistics/SRS_Report_2018/SRS_Statistical_Report_2018.pdf) estimates India's CDR at 6.2 per 1K, while this drops to 6.0 per 1K in the 2019 report. Using population projections [4], the SRS estimates of CDR imply 92–96% completion nationally in 2019. There are, however, several reasons to believe that the estimates of CDR at 6.0–6.2 could be too low, and hence the estimate of 92–96% completion too high.

*UN estimates*. The United Nations estimated CDR in India to be 7.2 per 1K for the period 2015–20 [5], based on methodologies described by Gerland 2014 [6]. This would imply an estimated registration completion during 2019 of only 80%.

*Combining sub-national estimates*. We can combine sub-national estimates of registration completion in the 2019 CRS report to estimate national completion, and hence CDR. These calculations cap registration completion at 100%, i.e., if a region saw more deaths registered than expected from its estimated CDR, then registered deaths are taken as an estimate of total deaths. In each region we use the completion estimate to estimate total deaths, and summing these gives an estimated national death toll in 2019; from this we obtain national estimates of CDR and completion. This process gives an estimated national CDR in 2019 of 6.6 per 1K, and registration completion of around 86%. These estimates are based on subnational estimates of CDR from the 2018 SRS report. If, instead, we use data from the 2019 SRS report, then we obtain an estimated national CDR in 2019 of 6.5 per 1K, and registration completion of around 88%.

*Estimates based on age-stratified mortality rates*. The 2018 and 2019 SRS reports give estimated death rates in different age groups. Projected population pyramids are available for 2016 and 2021, but not intervening years [4]. Using the estimated age-specific mortality rates in the SRS reports, and the projected 2016 age distribution, we obtain an estimated national CDR of 6.6–6.8 per 1K, which would imply registration completion in 2019 of 84–87%. Using, instead, the 2021 age distribution, we obtain an estimated CDR of 7.3–7.5 per 1K, implying registration completion in 2019 of 76–79%. (Estimates depend on the fraction of the over-80s who are assumed to be over 85, which is not given in the projected population pyramids; we set this to be 0.375 as estimated in both SRS reports.)

*Estimates based on NFHS-5*. NFHS-5 interviews were conducted between 2019 and 2021 (Government of India 2021) [7]. The survey asked respondents about deaths of any usual family member in the previous few years, and whether the death was registered. From this data, we can compute a national registration level for 2018 of 73%, and hence a CDR for 2018 of 7.2. Assuming no change in the CDR between 2018 and 2019, we get estimated registration completion in 2019 of around 79%.

We thus find estimates of CDR nationally ranging from 6.0 to 7.5, corresponding to registration completion in 2019 ranging from 76% to 96%, with a cluster of estimates around 80%. The estimated completion of 86% derived from sub-national data in the 2019 CRS report may thus somewhat overestimate registration completion in India. We return to this point when discussing our results. More detail and full calculations are available at IndiaCOVIDmapping.org [8].

Figure A1 shows that for most states, levels of death registration completion observed in the NFHS-5 are lower than estimated official levels of death registration completion. The most likely explanation of over-estimation of official levels death registration completion is that the Crude Death Rate, estimated by the SRS, is under-estimated. When compared to official population projections and the 2011 Census, the SRS underestimates the proportion of people above age 65, and this contributes to lower Crude Death Rates in the SRS.


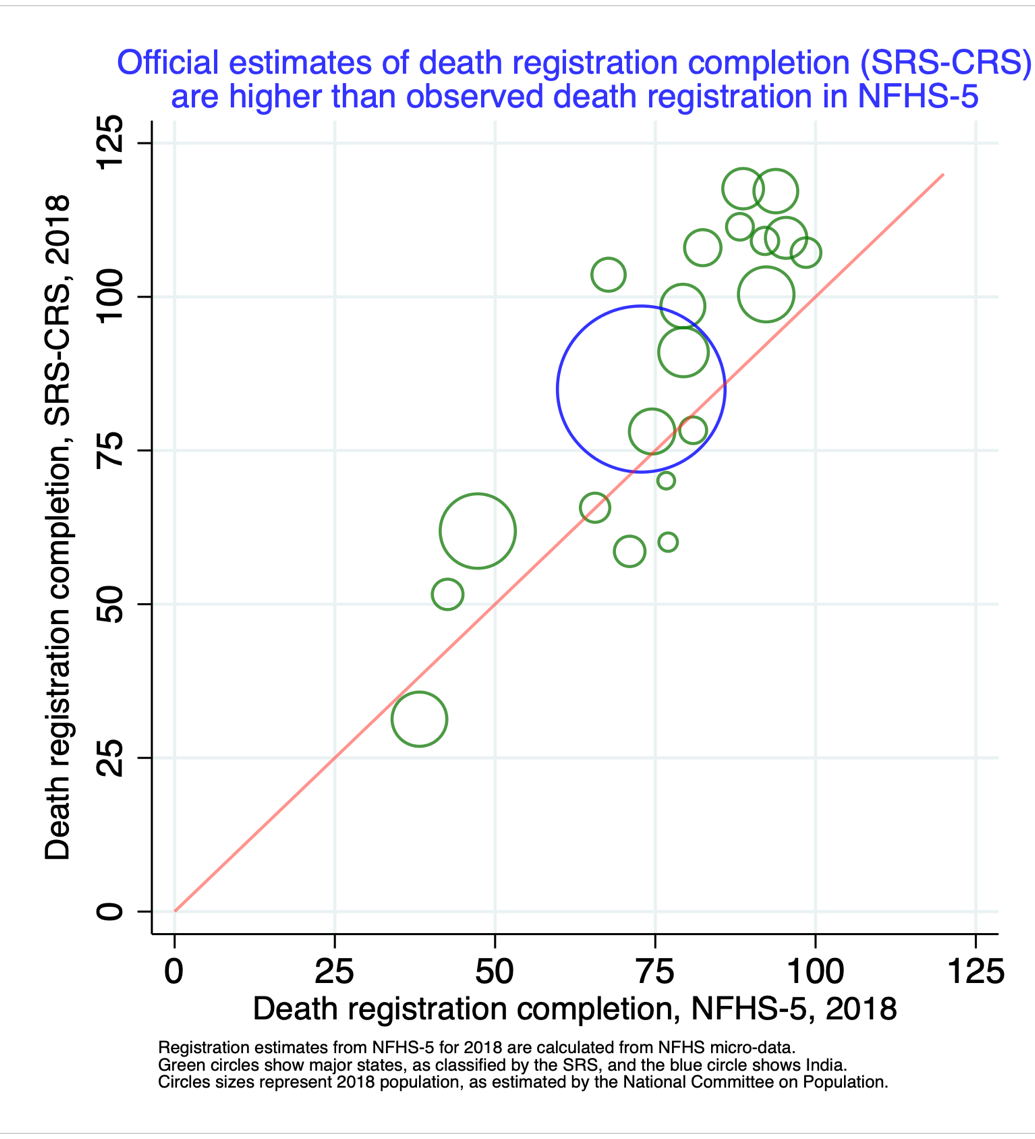


**Fig A**: Comparison of estimates of death registration completion

**Computing excess deaths, and sensitivity of the estimates to parameter changes**

In order to estimate excess deaths in a given location over a given period, we need to estimate expected deaths and actual deaths. Given death registration data during some pandemic period and some comparable reference period, we additionally need estimates of:

1. coverage in the data during the reference period
2. coverage in the data during the pandemic periodic
3. expected changes in mortality between the reference period and pandemic period

With quantities in appropriate units, we can then compute:

1. (Pandemic deaths) = (pandemic registrations) / (pandemic coverage)
2. (Baseline deaths) = (baseline registrations) / (baseline coverage)
3. (Expected deaths) = (expected change) * (baseline deaths)
4. (Excess deaths) = (pandemic deaths) ­− (expected deaths)
5. P-score = 100*(excess deaths) / (expected deaths)

Finally, if we wish to extrapolate from some regions to others, we also need estimates of how the mortality impact may have varied between the regions for which we have data, and those for which we don't.

We can examine how small changes in the key parameters affect our central estimates of excess deaths in STAR12 during April 2020–May 2021, and nationally. For each state, we make a small percentage change in each parameter of interest, namely baseline coverage in the data, baseline mortality, pandemic-period coverage in the data, and the difference in mortality impact in regions other than STAR12. The resulting outputs (excess deaths per capita and P-scores) are then compared with their baseline values. Calculations are available online [9]. From this process, we obtain the following estimates.

- *Changes in coverage*. A 1% (relative) decrease in pandemic period coverage in the data in each state relative to 2019 causes a 4.6% increase in excess deaths estimates in STAR12. Based on pre-pandemic trends and the disruption we see early in the pandemic, we might optimistically hope to see a relative increase in coverage in the data of 5% in states where this is possible; pessimistically, we might expect a relative decrease in coverage in the data of 5%. The reality is most likely that the competing effects of disruption and recovery summed in different ways in different states.
- *Changes in expected deaths*. A 1% increase in expected deaths during the pandemic period relative to 2019 (as a consequence of population growth and changes in CDR) would cause a 3.6% decrease in excess mortality estimates in STAR12. Based on pre-pandemic population growth and trends in CDR, we might expect year-on-year deaths to remain fixed or rise by at most 2%.
- *Errors in estimation of baseline mortality/coverage*. A 1% (relative) decrease in both baseline and pandemic period coverage in the data in each state causes a 1% increase in excess mortality estimates in STAR12. Optimistically, we might hope that the sub-national estimates of registration completion in the 2019 CRS report are accurate. A more pessimistic view would be that in some states they could have been overestimated by up to 15%.
- *Difference in mortality impact outside STAR12*. If regions not in STAR12 collectively saw a mortality impact 1% higher than in STAR12, this causes a 0.4% increase in the national mortality estimates. We consider it plausible that the mortality impact in STAR12 could differ by up to 20% from that in the remainder of the country, causing an 8% shift in our excess mortality estimates.

When we consider the effects and possible scale of the uncertainties in these parameters, it is clear that shifts in coverage in the data during the pandemic cause the greatest uncertainty in the estimates of excess mortality. By comparison, the likely effects of population growth, changes in CDR, and errors in estimates of pre-pandemic registration coverage are relatively small. There is also no compelling reason to believe that the mortality impact of the pandemic outside of STAR12 should have been considerably different from that in STAR12.

**SI References**

1. Government of India. Vital Statistics of India Based on the Civil Registration System 2019. New Delhi: Office of the Registrar General of India, Ministry of Home Affairs, Government of India. 2021.

2. ORGI. Sample registration system statistical report 2018. Office of the Registrar General and Census Commissioner of India, New Delhi: Government of India.; 2020.

3. ORGI. Sample registration system statistical report 2019. Office of the Registrar General and Census Commissioner of India, New Delhi: Government of India.; 2022.

4. National Commission on Population. Population Projections for India and States 2011-2036. Report of the Technical Group on Population Projections Constituted by the National Commission on Population. 2019.

5. United Nations, Department of Economic and Social Affairs, Population Division. World Population Prospects 2019 Data Booklet.

6. Gerland P. UN Population Division’s methodology in preparing base population for projections: case study for India. Asian Population Studies. 2014;10: 274–303.

7. International Institute for Population Sciences (IIPS), ICF. National Family Health Survey (NFHS-5), 2019-21: India. Mumbai: IIPS; 2022.

8. India COVID Mapping. Mapping the impact of the COVID-19 pandemic in India. In: https://www.indiacovidmapping.org/ [Internet]. 2022. Available: https://www.indiacovidmapping.org/

9. Banaji M. IndiaACMdata/National Estimates. In: GitHub [Internet]. 2022 [cited 29 Sep 2021]. Available: https://github.com/muradbanaji/IndiaACMdata/tree/master/NationalEstimates

Legends

Fig A: Comparison of estimates of death registration completion
